# ERP effects at encoding: Image memorability or recognition success?

**DOI:** 10.3758/s13415-026-01427-z

**Published:** 2026-04-21

**Authors:** Will Deng, Kara D. Federmeier

**Affiliations:** 1 Department of Psychology, University of Illinois Urbana-Champaign, 603 E Daniel St., Champaign, IL 61820 USA; 2 Program in Neuroscience, University of Illinois Urbana-Champaign, Champaign, IL USA; 3 Beckman Institute for Advanced Science and Technology, University of Illinois, Champaign, IL USA

**Keywords:** ERP, Memorability, Episodic memory, Semantics

## Abstract

The subsequent memory effect (SME) refers to neural patterns (e.g., in EEG or fMRI) at encoding that predict later memory performance. In N400-based SMEs, for example, items later remembered elicit less negative N400 amplitudes at encoding compared to items later forgotten. These effects have traditionally been interpreted as reflecting idiosyncratic neural states during encoding—in the case of the N400, states related to semantic activation—that influence episodic encoding success. However, recent work on memorability, a stable, item-level property indicating the population-level likelihood that an image will be remembered, has shown that high (compared to low) memorability images elicit less negative N400 amplitudes, suggesting that memorability is linked to more targeted semantic mapping. This raises the question of whether encoding-related effects are more tied to intrinsic stimulus properties or in-the-moment encoding variability. The present study examined both factors in tandem: ERPs were recorded while participants viewed images varying in memorability and were later classified by recognition outcome (hit vs. miss). Analyses revealed that N400 amplitudes were significantly predicted by memorability scores even when controlling for subsequent memory performance. Memorability also predicted Late Positive Complex SMEs. These findings suggest that neural activity traditionally associated with later memory success may capture item-level properties rather than transient encoding states. Consequently, memorability appears to be a key driver of differences in memory performance, challenging interpretations of SMEs as purely state-dependent and highlighting the importance of considering intrinsic stimulus characteristics when evaluating effects correlated with memory success.

Memorability refers to a stable, item-level property of images that reflects the likelihood that a given image will be recognized when encountered again (Bainbridge et al., 2013; Bylinskii et al., 2015; Isola et al., 2014; Khosla et al., 2015). It has often been estimated with a continuous recognition task wherein participants view a series of new and repeated images with delayed repetition and identify the repeats. A memorability score is calculated as the average hit rate or *d*-prime (Bainbridge, 2017; Bainbridge & Rissman, 2018) for each item’s repetition, such that items with higher memorability are those that are more likely to be remembered at the group level. A defining feature of memorability is its consistency: Rank-order differences among images are highly reproducible across individuals, task contexts, and testing conditions (Bainbridge et al., 2013; Isola et al., 2011), and memorability can be predicted from (decontextualized) image content alone using convolutional neural network models (Khosla et al., 2015; Needell & Bainbridge, 2022). Although recognition performance in any given instance is influenced by factors such as encoding depth, attention, and context, rank order memorability has been found to be fairly stable across these varying levels of performance (Bainbridge, 2020; Bylinskii et al., 2015), such that memorability can be seen as capturing variance that is shared across observers at the level of the stimulus itself.

The underlying source(s) of memorability differences across images have proven to be difficult to identify. Various factors, such as low-level visual properties, including color, hue, and spatial frequency (Bainbridge et al., 2017; Bylinskii et al., 2015; Isola et al., 2014), subjective ratings of image aesthetic or interestingness (Isola et al., 2014), and cognitive factors related to depth of encoding or attention (Bainbridge, 2020; Wakeland-Hart et al., 2022), have been examined and found not to strongly predict variance in memorability. In previous work, we probed for the functional underpinnings of memorability using event-related potentials (ERPs) time-locked to image onset recorded in a continuous recognition task with delayed repetition (Deng et al., 2024a). We focused on the N300 and N400 components, which are linked to high-level visual processing and long-term semantic memory access, respectively. The N300 is a frontally distributed, negative-going component that peaks around 300 ms, observed to visual stimuli such as line drawings and photographs of objects (Barrett & Rugg, 1990; McPherson & Holcomb, 1999), faces (Jemel et al., 1999), and scenes (Kumar et al., 2021). It has been linked to visual template matching (Schendan & Lucia, 2010; Kumar et al., 2021), as its amplitude is smaller (less negative) for images that are easier to recognize and/or categorize (Schendan & Kutas, 2002). The N400 is a negative-going component that peaks around 400 ms and that is observed to potentially meaningful stimuli of all types in all modalities, including words and sounds (Kutas et al., 1987; Van Petten & Rheinfelder, 1995), as well as line drawings, photographs, and movies (Ganis et al., 1996; Holcomb & McPherson, 1994; McPherson & Holcomb, 1999; Sitnikova et al., 2008). The N400 has been argued to index access to long-term semantic memory, with smaller (less negative) amplitudes for stimuli that elicit less new semantic information (reviewed in Kutas & Federmeier, 2011; Federmeier, 2022). Stimuli that are more confusable with others and/or not reliably comprehended may not be efficiently mapped onto specific semantic information and hence yield more net activity across semantic memory: Unrecognizable objects (Supp et al., 2005), improbable images (Proverbio & Riva, 2009), and wordforms that have high similarity to other words (large orthographic neighborhoods; Laszlo & Federmeier, 2011) elicit larger N400 responses.

The ERP results revealed memorability effects on both the N300 and N400 and suggested that memorability may reflect processing at the interface between perceptual analysis and semantic activation. High memorability images elicited smaller amplitude N300 and N400 responses even at just the first presentation (Deng et al., 2024a). The smaller N300 amplitudes for high memorability images support the idea that these images enjoy facilitated template matching, which aids in recognition/identification (Schendan & Kutas, 2002; Kumar et al., 2021). This is consistent with findings in a previous study showing that high memorability images are more readily perceived (Deng et al., 2024b). The smaller N400 amplitudes for high memorability images support the idea that they are more precisely mapped onto semantic features, leading to an overall lower level of semantic activation (Kutas & Federmeier, 2011; Federmeier, 2022). In turn, in the context of memory, reduced N400s have been linked to higher conceptual fluency, which leads to better recognition (Hou et al., 2013; Voss et al., 2010; Wang et al., 2015).

Crucially, the semantic activation reflected by the N400 at first presentation seems to serve an important role in mediating how images are processed at second presentation, when recognition occurs. When the images were repeated, we observed repetition effects (less negative amplitudes at second presentations compared to the first) for both the N300 and N400, consistent with previously established patterns in old/new recognition paradigms (Eddy et al., 2006; Rugg & Curran, 2007). The magnitude of the repetition effect can indicate the degree to which exposure at the first presentation has facilitated the processing of visual representations and semantic information at the second presentation (Schendan & Maher, 2009; Voss & Paller, 2007). We found no differences in the size of N400 repetition effects as a function of memorability, but N300 repetition effects were larger for high memorability items, suggesting enhanced perceptual fluency for these images following initial exposure. Strikingly, the size of the N300 repetition effect was predicted by N400 amplitudes at the initial presentation. We interpreted this pattern as indicating that more precise semantic activation when an image is first encountered can help to refine the perceptual templates used to identify that image (Deng et al., 2024a). This points to the possibility that semantic activation at the first exposure can have a lasting impact on how images are later perceived and remembered.

Brain activity (EEG or fMRI-based) patterns when a stimulus is first encountered that predict later memory have also been studied in the context of what are known as Subsequent Memory Effects (SMEs) or Difference due to memory (Dm) effects (Forester & Kamp, 2023; Packard et al., 2017; Sanquist et al., 1980; Van Petten & Senkfor, 1996; Yovel & Paller, 2004). By back-sorting trials during the encoding phase based on whether a stimulus is later remembered or forgotten, researchers have been able to isolate brain activity predictive of later memory-based behavioral patterns (reviewed in Mecklinger & Kamp, 2023). SMEs in EEG data have been characterized for different types of stimuli, including words (Fernandez et al., 1998; Packard et al., 2017), objects (Duarte et al., 2004; Kamp & Zimmer, 2015), scenes (Gutchess et al., 2007; Höltje & Mecklinger, 2020), and faces (Guo et al., 2005; Yovel & Paller, 2004). The ERP patterns associated with SMEs are varied and can encompass a relatively wide range of processing time windows, with some starting as early as 300 ms post-stimulus-onset (Meng et al., 2014) and others starting as late as 1,400 ms (Kamp & Zimmer, 2015). These effect patterns have provided indications of some of the factors that contribute to memory performance. For example, stimuli that elicit enhanced P300s at encoding tend to be better recalled, and this SME has been interpreted as reflecting attention-based processes associated with distinctiveness (Kamp & Donchin, 2015; Wiswede et al., 2006; although see Fabiani et al., 1990, for the effect of encoding strategies on P300 SMEs). SMEs based on later components, such as the Late Positive Complex (LPC; seen after 500 ms post-stimulus-onset), with enhanced LPC activity at study predicting better memory performance, have been interpreted as reflecting the positive impact of retrieval practice on recollection (Jia et al., 2021; Liu et al., 2017).

Studies looking at N400-based SMEs have investigated the impact that semantic processing in particular can have on later memory. For example, when participants were asked to recall previously studied words, the successfully recalled words were found to have elicited less negative N400s at encoding compared to those that were not recalled (Fernandez et al., 1998). Similarly, stimuli that were later successfully recognized have been found to elicit reduced N400 amplitudes at encoding compared to those that failed to be recognized (Van Petten & Senkfor, 1996). Semantic activation patterns have been shown to not only contribute to memory performance but to also moderate memory-based differences. In some studies, participants learned words that were preceded by either a semantically congruent cue (e.g., *apple* cued by *fruit*) or an incongruent cue (e.g., *bear* cued by *metal*). It is well established that N400 amplitudes to targets are reduced when they are congruently, compared to incongruently, cued (Federmeier et al., 2010). Interestingly, cuing interacts with N400 SMEs, such that the pattern of less negative amplitudes for subsequent hits versus misses was observed only when words were congruently cued, whereas words paired with incongruent or neutral cues did not elicit an N400 SME (Höltje et al., 2019; Meßmer et al., 2021; Packard et al., 2017). Similarly, when participants read sentences ending with either expected or unexpected words, the expected words elicited a more prominent SME than did the unexpected words (Höltje & Mecklinger, 2022).

Because N400 amplitudes have been understood to reflect the amount of semantic information elicited by a stimulus (Federmeier, 2022; Kutas & Federmeier, 2011), the reduced N400 amplitudes elicited by words that are more likely to be recognized or recalled in the SME studies suggests that more targeted semantic activation can yield memory benefits. Congruent words or sentence contexts can help to guide semantic activation in a way that is beneficial to downstream memory. Even without cuing, however, variation in semantic processing can be predictive of later memory, suggesting that semantic processing and memory encoding are intertwined.

Notably, then, less negative N400 responses at encoding have been associated both with better subsequent memory performance, as sorted within individual participants, as well as with higher memorability, defined at the level of the stimulus. This raises the question of whether the observed patterns capture neural states promoting in-the-moment episodic encoding, which may be idiosyncratic across people (as SMEs have typically been interpreted), or, instead, intrinsic properties of the stimuli that make some of them more likely to be recognized across different people (as memorability effects have typically been defined). Previous fMRI and MEG work has shown that item-driven memorability effects can be separated from effects of individual memory performance (Bainbridge et al., 2017; Bainbridge & Rissman, 2018; Mohsenzadeh et al., 2019). We sought to perform a similar analysis with ERPs, focusing on whether the amount of semantic activation indexed by the N400 at the first presentation is more yoked to memorability (independent of individual memory success) or, instead, to subsequent memory success (once memorability is controlled).

In our previous study, participants had very high memory performance (hit rates of 0.94 for high and 0.89 for low memorability images) due to the short lag implemented between the first and second presentations in the continuous recognition task, making it difficult to include participant performance in our analysis (Deng et al., 2024a). Therefore, in the current study, we lengthened the delay between the first and second presentations by separating the study and test phases across 2 days to allow for more behavioral variation and thereby allow us to examine how ERP responses at initial presentation relate both to image memorability and to later recognition performance. We expected to replicate our previous N300 and N400 patterns at the first presentation, wherein high memorability images elicit less negative amplitudes compared to low memorability images. We also expected to see a general N400 SME, such that N400 amplitudes would be less negative for items that were subsequently recognized compared to those that were forgotten. The critical question concerns how these two sources of variance relate to one another when considered jointly. Previous fMRI and MEG findings suggest that we could find separable effects of individual memory performance and memorability, because these have been linked to differentiable brain networks (Bainbridge et al., 2017; Bainbridge & Rissman, 2018; Mohsenzadeh et al., 2019). At the same time, it remains possible that a substantial portion of variance previously attributed to in-the-moment encoding success, particularly in N400-based SMEs, instead reflects the kind of stable, item-level differences that drive memorability. Accordingly, the present study was designed to assess the extent to which N400 variance at encoding is accounted for by item-level memorability and to determine whether recognition success contributes additional explanatory power beyond memorability when both factors are considered together.

In addition to the N300 and N400 components, we also measured the late positive component (LPC). As already mentioned, the LPC is a later (500–800 ms) positive-going component with a posterior distribution that has been associated with explicit recollection (Curran, 2000; Finnigan et al., 2002) and recognition confidence (Addante et al., 2012). More positive LPC amplitudes when a stimulus is presented for the first time have been suggested to index high encoding fluency and shown to predict better downstream memory performance (Mecklinger & Kamp, 2023). The focus of our analyses on this component will again be to delineate the relative contribution of memory performance and stimulus memorability to observed encoding effects.

## Methods

### Participants

A target sample size of 24 was determined based on the sample size in our previous study using similar materials and procedures (Deng et al., 2024a). To obtain the target sample size, we recruited 32 participants from the University of Illinois who were paid for their time. Participants all self-reported to be right-handed, have normal or corrected-to-normal vision, and no history of head trauma, seizures, or neurological or reading disorders. Written informed consent was obtained in accordance with procedures and protocols approved by the University of Illinois Institutional Review Board. Data were excluded from five participants who did not show up to the second part of the experiment (see *Procedures*) and from three participants for poor data quality, leaving 24 participants (19 self-identifying as female and 5 as male, *M*_*age*_ = 19.88) for analysis.

## Materials

**Fig. 1:**
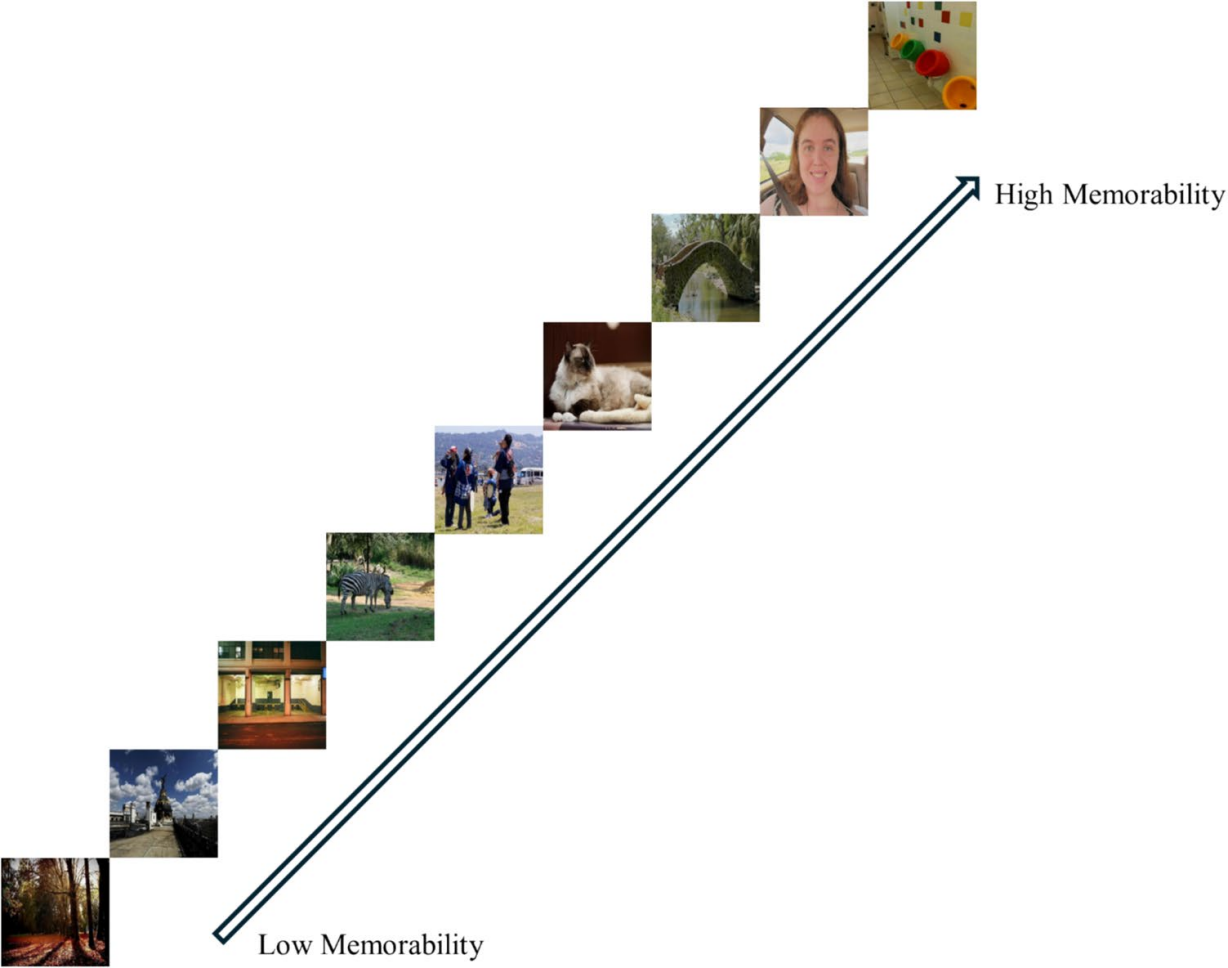
Image examples arranged based on their memorability scores (*M* = 0.71, *SD* = 0.25, range = 0.2–1.0)

A total of 375 images were obtained from the LaMem dataset (Khosla et al., 2015), 75 each from five experimenter-determined categories (animal, architecture, nature, object, and people). Memorability scores (*M* = 0.71, *SD* = 0.25, range = 0.2–1.0) in the dataset came from a norming experiment and consisted of the average hit rates for image recognition upon second presentation. Figure 1 shows examples of the images arranged based on their memorability scores. For the test phase, 125 new images were selected from the same dataset, again with equal distribution across the five stimulus categories (*M* = 0.73, *SD* = 0.2, range = 0.23–1.0). All images were resized to 512 x 512 pixels.

## Procedures

### Study phase

Participants were instructed to respond to a series of images by pressing one of four buttons with their right hand on a Cedrus RB-830 Response Pad (Cedrus, USA) to indicate if they had “low confidence,” “somewhat low confidence,” “somewhat high confidence,” or “high confidence” that they would remember an image later. The stimuli were presented on a 60-Hz CRT monitor of resolution 1280 x 1024 with the PsychoPy 2022.2.4 package (Peirce et al., 2019) and Python (Python Software Foundation, Python Language Reference, version 3.6.6). Participants’ EEG was recorded as they sat in a sound-attenuated recording booth, 118 cm away from the monitor, and were asked to remain still and to minimize eye movements and blinks during stimulus presentation. On each trial, a blue fixation cross at the center of the screen was displayed for 1,000 ms against a black background, followed by an image for 1,000 ms, and then another white fixation cross for 2,000 ms. Participants were allowed to respond as soon as an image appeared and before a blue fixation cross showed up for the next trial. Participants completed a 30-trial practice before the main experiment, which consisted of 375 trials divided into three blocks, with the block order counterbalanced across participants. The images in the practice trials did not overlap with the images in the main experiment.

### **Test phase**

The test phase occurred two days after the study phase in the same recording booth. Participants were informed that they would see a series of images, some of which they might have seen before during the study phase. They were then instructed to press a button with their right index finger if an image was new and to press a different button with their right middle finger if they had seen an image before. The images were presented the same way as in the study phase, and participants used the same response pad. No EEG was recorded in the test phase. Participants completed a 30-trial practice (old items were sampled from the practice trials in the study phase) before the main experiment, which consisted of 500 trials (375 images previously presented in the study phase and 125 new filler images) divided into five blocks, with the block order counterbalanced across participants.

## EEG recording parameters

**Fig. 2:**
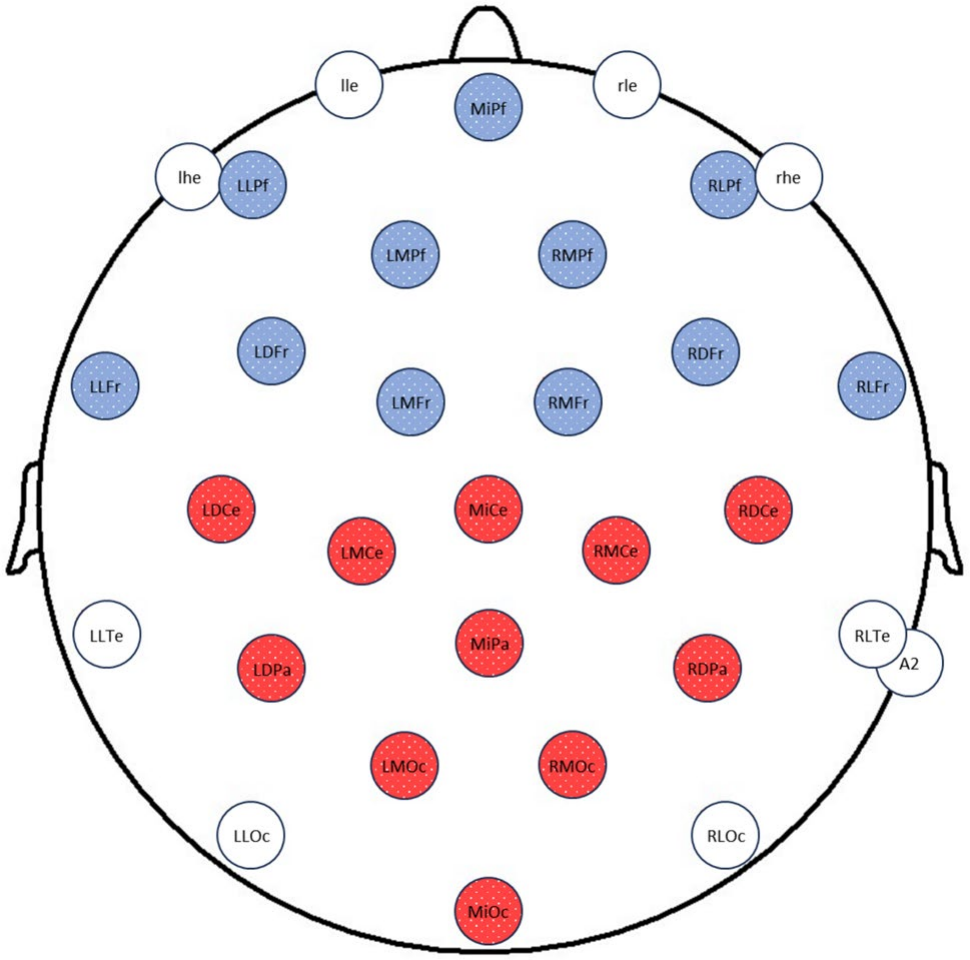
Electrode layout with frontal and central-posterior ROIs. Blue circles indicate channels in the frontal ROI, and red circles indicate channels in the central-posterior ROI

During the study phase, we recorded continuous EEG using 26 silver/silver-chloride electrodes spread evenly over the head and fixed in an elastic cap. The signals were amplified with a BrainAmpDC amplifier (Brain Products, USA). The 26 electrodes were placed at locations over midline prefrontal (MiPf), left and right medial prefrontal (LMPf and RMPf), left and right lateral prefrontal (LLPf and RLPf), left and right medial frontal (LMFr and RMFr), left and right mediolateral frontal (LDFr and RDFr), left and right lateral frontal (LLFr and RLFr), midline central (MiCe), left and right medial central (LMCe and RMCe), left and right mediolateral central (LDCe and RDCe), midline parietal (MiPa), left and right mediolateral parietal (LDPa and RDPa), left and right lateral temporal (LLTe and RLTe), midline occipital (MiOc), left and right medial occipital (LMOc and RMOc), and left and right lateral occipital (LLOc and RLOc) areas (Fig. 2). We also placed electrodes on the outer canthus and infraorbital ridge of each eye to record blinks and saccades. Recordings were referenced online to the left mastoid and re-referenced offline to the average of the left and right mastoids. Electrode impedances were kept below 5 kΩ. EEG was digitized with a 0.02–250 Hz band pass and a sampling rate of 1,000 Hz.

### ERP data processing and analysis

Trial-level EEG data were processed with EEGLAB (Delorme & Makeig, 2004) and ERPLAB (Lopez-Calderon & Luck, 2014) toolboxes in Matlab. Each trial was a 1,100 ms epoch from −200 to 900 ms time-locked to study phase presentation onset and baseline corrected with the mean amplitude of the 200 ms window prior to stimulus onset. We applied a 30 Hz low-pass filter and did artifact rejection for blinks, saccades, drift, and excessive muscle activity. Blinks and saccades were identified using thresholds calibrated for each participant in a condition-blind manner using visual inspection. Independent component analysis using AMICA was applied to participant data if there were blinks in more than 25% of the epochs (N = 7; Delorme et al., 2012); in this case, components were removed if they correlated with the eye channels at above 0.5. Epochs with artifacts that were not corrected were removed from all participant EEG data. Using the same exclusion criteria as our previous study that we aimed to replicate (Deng et al., 2024a) and following general recommendations for ERP research (Luck, 2014), participants with greater than 25% of epochs removed due to artifacts even after applying ICA were excluded from analysis (*N* = 3). On average, 90% of trials were retained with a range of 78–100% across participants.

To examine N300 effects, which are known to peak around 300 ms and to be largest over the front of the head (Hamm et al., 2002; Kumar et al., 2021; Schendan & Lucia, 2010), we computed mean amplitudes for each trial in an *a priori* 250–350 ms time window over all 11 frontal channels (MiPF, LLPf, RLPf, LMPf, RMPf, LLFr, LDFr, LMFr, RMFr, RDFr, RLFr; Fig. 2). N400 effects to pictures also show a frontally skewed distribution (Federmeier & Kutas, 2002; Ganis et al., 1996), so we used this same frontal region of interest (ROI) to characterize the N400, measuring mean amplitudes in an *a priori* 350–500 ms window. To examine the LPC effect, based on its typical distribution in the literature (Curran, 2000; Finnigan et al., 2002), we used an 11-channel central-posterior ROI (LDCe, LMCe, MiCe, RMCe, RDCe, LDPa, MiPa, RDPa, LMOc, MiOc, RMOc; Fig. 2) in a time window of 500–800 ms.

To confirm whether the current study replicated our prior findings, we first adopted a dichotomized image memorability condition (high vs. low memorability) based on a median split of image memorability scores. The single trial amplitude means were then fitted to a linear mixed-effect model with a dummy-coded categorical effect of memorability, random intercepts for participants and images, and random slope of memorability for participants. To check whether we obtained a general SME, the single trial means were fitted to a linear mixed-effect model with a dummy-coded categorical effect of downstream memory performance (hit vs. miss), random intercepts for participants and images, and random slope of memory performance for participants. For models investigating SME and memorability together, we retained the continuous memorability scores for the memorability condition. The single trial means were fitted to linear mixed-effect models with a continuous fixed effect of memorability and a dummy-coded categorical fixed effect of memory performance (hit vs. miss), random intercepts for participants and images, and random slope of memorability for participants (Bates et al., 2014). Significance testing of fixed effects was estimated with *t*-tests using the Satterthwaite method in the lmerTest package in R (Kuznetsova et al., 2017).

For the test phase recognition data, to verify the memorability effect and to examine the relationship of memory performance with participants’ study phase confidence judgments, we constructed a mixed-effect logistic regression model with the memorability score and dichotomized confidence judgements (high vs. low confidence) as fixed effects, random intercepts for participants and images, and random slopes of memorability score and confidence judgment for participant.

## Results

### Behavioral results

During the test phase, participants recognized images they had seen before at an average rate of 0.55 (*SD* = 0.13, range = 0.26–0.77) with a false alarm rate of 0.22 (*SD* = 0.14, range = 0.03–0.66). Normed continuous memorability scores reliably predicted image recognition (*z* = 9.17, *p* < 0.001) with the odds ratio suggesting that an increase of 0.10 in memorability score increases the odds of recognizing an image by 1.4. Participants were also able to predict their own memory performance (*z* = 6.60, *p* < 0.001), with high confidence ratings corresponding to an increased odds of recognizing an image by 1.93 compared to low confidence ratings. However, the confidence ratings were not highly correlated with memorability scores: Spearman’s rank correlation *ρ* = 0.17. The average memorability score was 0.78 (*SD* = 0.06) when participants gave high confidence ratings, and 0.68 (*SD* = 0.03) when participants gave low confidence ratings.

### ERP results

**Fig. 3:**
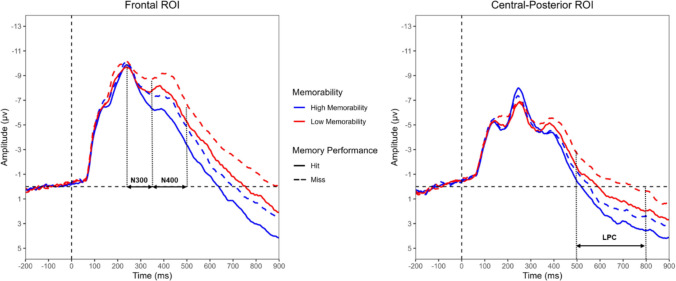
Grand average ERPs at the frontal and central-posterior ROIs. ERP responses are plotted to images divided by image memorability (high vs. low) and participant memory performance (hit vs. miss) at test. For illustrative purposes, images are halved into high and low conditions based on their memorability scores. Time windows used to assess the N300, N400, and LPC are marked

**Fig. 4:**
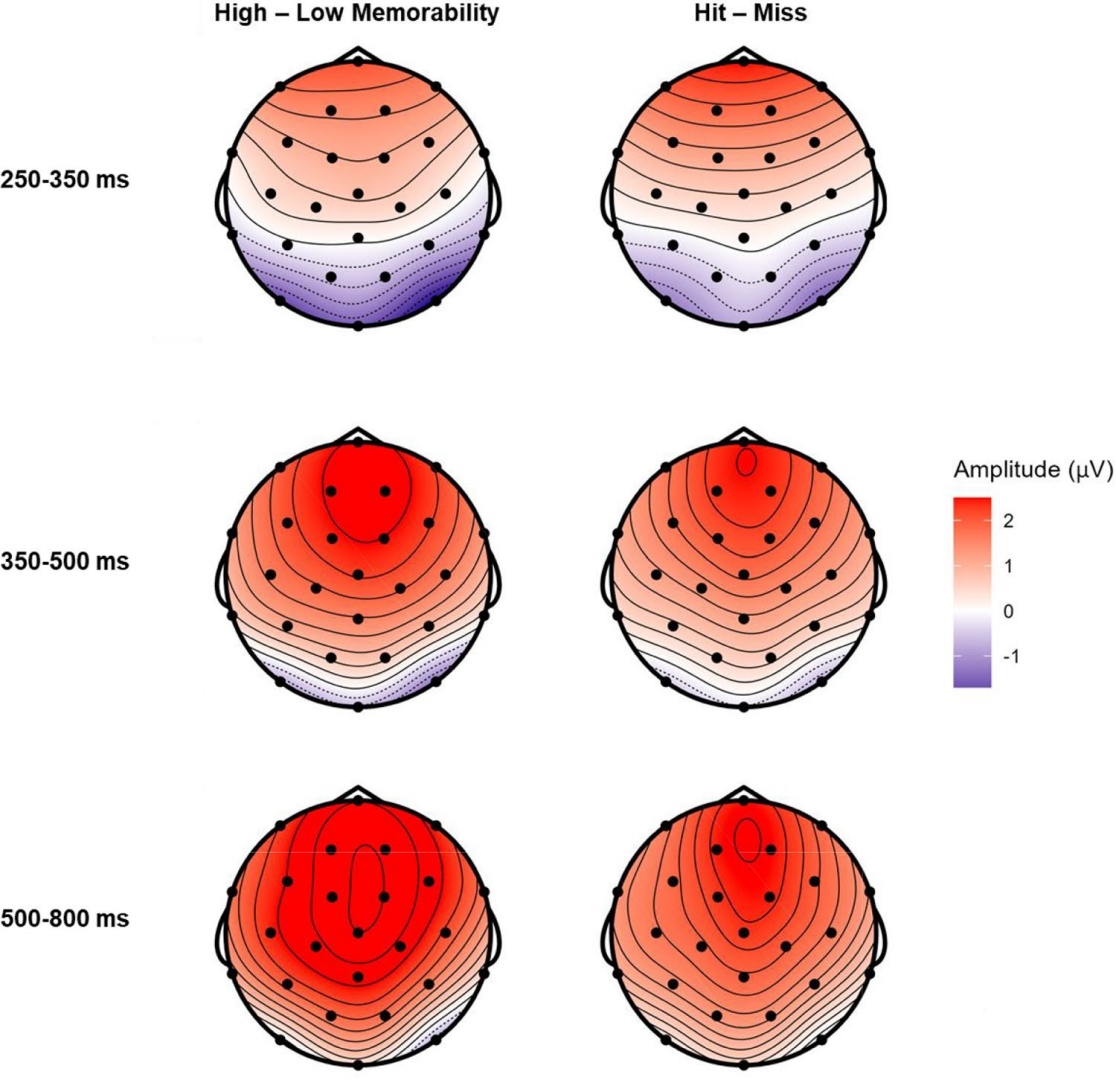
Topographical maps show the scalp distribution of the memorability effect (high - low) in the left column and of the SMEs (hit - miss) in the right column. For illustrative purposes, images are halved into high and low conditions based on their memorability scores. The rows show the distribution in the N300, N400, and LPC time windows

Figure 3 shows the frontal and central-posterior grand average ERPs grouped by memorability and whether or not images were recognized at test. Across all three time windows of interest (N300, N400, LPC), responses were most positive to high memorability hits and most negative to low memorability misses. Figure 4 shows the scalp distribution of the memorability and SMEs across the three time windows. We first assessed memorability and SMEs separately and then modeled the two together. On average, when memorability is dichotomized, 169 (range: 147–187) trials contributed to high memorability bins and 166 (range: 137–188) to low memorability bins; for SMEs, 186 (range: 85–274) trials contributed to remembered bins and 150 (range: 71–260) to forgotten bins. Within high memorability, 0.66 (range: 0.34–0.85) of the trials were later remembered, and within low memorability, 0.44 (range: 0.15–0.71) of the trials were later remembered.

#### **Memorability**

A mixed-effect model for the N300 time window revealed a main effect of the dichotomized memorability condition (*t* = 2.65, *p* = 0.009), with high memorability images eliciting less negative amplitudes (−7.9 µV) compared to low memorability images (−8.67 µV). The same model for the N400 time window also revealed a main effect of memorability (*t* = 6.39, *p* < 0.001) with high memorability images eliciting less negative amplitudes (−5.69 µV) compared to low memorability images (−7.87 µV). The same effect was found for the LPC time window (*t* = 6.11, *p* < 0.001) with high memorability images again eliciting less negative amplitudes (2.07 µV) compared to low memorability images (−0.07 µV). The memorability differences for N300 (*t* = 2.01, *p* = 0.045), N400 (*t* = 5.30, *p* < 0.001), and LPC (*t* = 5.68, *p* < 0.001) remained when participant confidence (high vs. low) was included in the models. Thus, the present data replicated our prior findings (Deng et al., 2024a) of the effect of memorability on N300, N400, and LPC responses to initial picture presentation.

#### **Subsequent memory effects (SMEs)**

There was a general N400 SME (*t* = 3.56, *p* = 0.001), such that images that were subsequently recognized elicited less negative N400 amplitudes at encoding (−5.86 µV) compared to those that later failed to be recognized (−7.89 µV). There was also an LPC SME (*t* = 2.83, *p* = 0.009), such that images that were subsequently recognized elicited more positive LPC amplitudes at encoding (1.44 µV) compared to those that later failed to be recognized (0.49 µV). No significant SME was found for the N300 (*t* = 1.85, *p* = 0.07). Thus, we replicate prior findings (Fernandez et al., 1998; Jia et al., 2021) of reduced (less negative) N400s and enhanced (more positive) LPCs to items that go on to be recognized compared to those that are forgotten.

#### **Joint impact of memorability and subsequent memory**

**Fig. 5:**
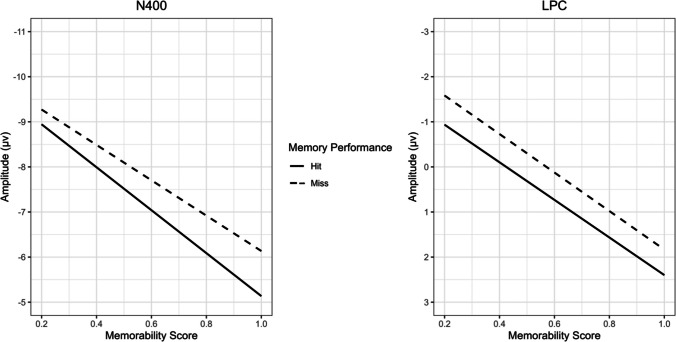
Model estimates of N400 and LPC amplitudes as a function of memorability score and performance

A mixed-effect model for the N300 time window with both continuous memorability scores and memory performance as fixed effects showed no main effect for either memorability (*t* = 1.76, *p* = 0.08; Table 1) or recognition (*t* = 0.24, *p* = 0.809) and no interaction between the two (*t* = 0.90, *p* = 0.367). However, the same mixed-effect model for the N400 time window revealed a main effect of memorability (*t* = 5.66, *p* < 0.001; Table 2) with a 0.1 increase in memorability score correlating to a 0.48 µV change in N400 amplitudes. In this model, with memorability accounted for, there was no significant main effect of recognition (*t* = 0.26, *p* = 0.794) or interaction between the two (*t* = 1.03, *p* = 0.303; Fig. 5). Similarly, for the LPC, the model revealed a main effect of memorability (*t* = 4.95, *p* < 0.001; Table 3) with a 0.1 increase in memorability score correlating to a 0.42 µV increase in LPC amplitudes but no main effect of recognition (*t* = 1.03, *p* = 0.301) or interaction between the two (*t* = 0.12, *p* = 0.907).

**Table 1:** N300 modeling results

Effect	Parameter estimate	Standard error	*t*-value	*p*
intercept	−9.16	0.99	−9.29	8.45E-12
memorability	1.36	0.77	1.76	0.080
recognition	0.14	0.59	0.24	0.809
memorability:recognition	−0.71	0.78	−0.90	0.367

**Table 2:** N400 modeling results

Effect	Parameter estimate	Standard error	*t*-value	*p*
Intercept	−9.90	1.03	−9.61	8.00E-12
Memorability	4.76	0.84	5.66	1.95E-7
Recognition	−0.16	0.61	−0.26	0.794
Memorability:recognition	−0.84	0.81	−1.03	0.303

**Table 3:** LPC modeling results

Effect	Parameter estimate	Standard error	*t*-value	*p*
Intercept	−1.77	0.79	−2.23	0.029
Memorability	4.17	0.84	4.95	3.17E-6
Recognition	−0.67	0.64	−1.03	0.301
Memorability:recognition	0.10	0.86	0.12	0.907

Although there was no main effect of recognition once memorability was in the same model, we wanted to probe further to see if including recognition performance did or did not significantly improve model fit. A likelihood ratio test between the original mixed-effect model and a nested model with only memorability scores as the fixed effect suggested that the addition of the recognition variable did improve model fit for both the N400, χ^2^(2) = 14.66, *p* < 0.001, and the LPC, χ^2^(2) = 7.55, *p* = 0.023. Thus, although much of the variance in N400 and LPC amplitude seems to be driven by memorability, there is evidence for an additional impact of encoding success on these component amplitudes.

## Discussion

Facilitated semantic processing, as indexed by reduced (less negative) N400 responses, has been linked to improved memory outcomes, both through subsequent memory effects (SMEs) and through effects based on stimulus-level memorability scores. The present study aimed to disentangle these factors to clarify the role of semantic processing in memory formation. In our previous ERP work, highly memorable images elicited attenuated N300 and N400 amplitudes during initial presentation, reflecting facilitated perceptual processing and semantic activation, respectively, and semantic processing at encoding was predictive of enhanced perceptual facilitation when the images were viewed a second time (Deng et al., 2024a). The idea that semantic processing is an important driver of memorability effects aligns with modeling studies indicating that semantic features account for a substantial portion of memorability variance (Kramer et al., 2023; Needell & Bainbridge, 2022). At the same time, N400-based SMEs have been reported in studies showing that subsequently remembered items elicit less negative N400 amplitudes than forgotten items (Fernandez et al., 1998; Packard et al., 2017; Van Petten & Senkfor, 1996). These findings raise a critical question: To what extent does variance in N400 activity during encoding reflect item-level properties captured by memorability scores versus (more idiosyncratic) in-the-moment episodic encoding processes that determine later memory success?

To address this question, we recorded ERPs while participants viewed images with varying memorability scores and then tested their recognition two days later. As discussed in more detail next, our results indicate that variance in ERP signals, on the N400 as well as the subsequent LPC, is largely accounted for by memorability, even when recognition performance is included in the model, with memorability emerging as the only significant fixed-effect predictor. This pattern suggests that a substantial portion of the attenuation observed in N400 amplitudes at encoding reflects item-level memorability differences that contribute to, but are not reducible to, later encoding success, underscoring the role of stimulus-driven factors in shaping both neural responses and memory outcomes.

We replicated our previous findings that high memorability images elicit less negative N300 and N400 amplitudes on first presentation compared to low memorability images (Deng et al., 2024a).^1^ This pattern reinforces the view that memorability reflects processes at the interface of high-level perceptual analysis and semantic activation, consistent with evidence suggesting that both perceptual and semantic features contribute to memorability variance (Needell & Bainbridge, 2022; Kramer et al., 2023). While memorability does not seem to be linked to variation in basic image features such as color and spatial frequency (Bylinskii et al., 2015; Isola et al., 2014) or to activation in early visual cortex (Bainbridge et al., 2017), it has been found to correlate with activation in areas associated with high-level perceptual processing, such as the parahippocampal place area and the lateral occipital complex (Bainbridge et al., 2017; Bainbridge & Rissman, 2018; Gu et al., 2023). High memorability images are also more readily perceived in visual detection tasks (Deng et al., 2024b), suggesting that they may contain perceptual features that better match stored perceptual templates, leading to more efficacious perceptual processing that aids in recognition (Beck et al., 2024; Center et al., 2024).

At encoding, however, semantic activation likely plays a more direct role in conferring the memory advantage. While facilitated perceptual processing may aid recognition by improving feature identification and categorization (Hamm et al., 2002; Kumar et al., 2021; Lamberts et al., 2002), facilitated access to semantic memory provides richer conceptual information that can guide recognition judgments. Indeed, modeling work suggests that semantic properties of an image contribute more to its memorability than do visual properties (“semantic primacy”; Kramer et al., 2023). Evidence from the current study further underscores this point: Whereas the memorability effect entailed both N300 and N400 differences, the subsequent memory effect was reliably observed only for the N400, with subsequent hits eliciting less negative amplitudes than misses. This suggests that downstream memory performance is more strongly linked to semantic activation at encoding than to high-level perceptual processing. An alternative possibility is that perceptual advantages emerge more fully upon re-exposure. As noted in the introduction, our prior work revealed larger N300 repetition effects for high-memorability images (with no corresponding N400 repetition differences), suggesting that perceptual fluency benefits may accumulate across presentations (Deng et al., 2024a).

The fact that N400 amplitudes can be predicted by both memorability and memory performance raises the question of which factor primarily drives these differences. Although higher memorability clearly contributes to better memory performance, as shown in our behavioral results, the neural mechanisms supporting these effects at encoding may differ. Previous fMRI findings have indicated an anterior shift in cortical sensitivity from memorability to memory performance, such that activation in the medial temporal lobe is more sensitive to memorability whereas activation in the prefrontal cortex is more sensitive to memory success (Bainbridge et al., 2017; Geissmann et al., 2023; Lahner et al., 2024). One interpretation is that frontal regions track individual variability in memory performance, whereas posterior regions, such as the lateral occipital cortex, are more closely tied to image-level properties. Because the N400 reflects activity in a distributed semantic network, including both the medial temporal cortex and inferior frontal cortex (Khateb et al., 2010; Lau et al., 2008; Tse et al., 2007), its amplitude may reflect the combined influence of both types of processes. Therefore, the semantic activation observed in our study could potentially represent a mixture of effects driven by intrinsic item-level properties (Deng et al., 2024a; Holcomb et al., 2002) and by variance due to in-the-moment encoding success (Fernandez et al., 1998; Van Petten & Senkfor, 1996).

One might reasonably assume that, when linking semantic activation to memory, N400 variance primarily reflects the degree of semantic activation unique to each individual during encoding, which in turn helps to determine whether an image will later be recognized, and that memorability serves only as a rough proxy for this relationship at the group level. However, our findings suggest something different. When both memorability and memory performance are included in the same model, higher memorability predicted less negative N400 amplitudes, whereas no significant main effect or interaction was observed for memory performance (even though, as discussed below, inclusion of memory performance does improve overall model fit). In other words, attenuation of semantic activation at encoding appears to be strongly influenced by image-level memorability. Although a general N400 SME emerges when memory performance is considered in isolation, a result that might typically be interpreted as evidence linking semantic activation to trial-level encoding success, the current results indicate that much of the N400 variance can be explained by intrinsic image properties that promote—but do not guarantee—encoding success.

The influence of image-level properties extends even to the late positive complex (LPC), which has traditionally been associated with explicit recollection performance (Curran, 2000; Finnigan et al., 2002; Mecklinger & Kamp, 2023; Neville et al., 1986) and which shows SMEs that are differentiable from those observed on the N400 (Hou et al., 2013; Olichney et al., 2000; Voss & Paller, 2007). As expected, we found an LPC-based SME, with later hits eliciting more positive amplitudes at the first presentation compared to later misses. However, when both memorability and memory performance were included in the LPC model, it was again only memorability that emerged as a significant predictor, such that higher memorability images elicited more positive LPC amplitudes (although, again, the inclusion of recognition did significantly improve model fit). This finding suggests that, beyond semantic activation, LPC-based processes during encoding are also shaped by intrinsic image properties, even when controlling for individual memory performance.

At the same time, the ERP patterns also indicate contributions beyond item-level memorability. As shown in Fig. 3, N400 and LPC amplitudes for both high and low memorability images appear to be attenuated when images are later forgotten, suggesting the presence of encoding-related modulation tied to individual memory success, an interpretation supported by the finding that inclusion of memory performance significantly improved model fit for both components, despite memorability accounting for the dominant share of variance. This contribution is worth noting, given that SMEs are expected to be more variable, because they are indexed by categorical trial-level outcomes derived from the current sample, whereas memorability scores reflect stable, continuous item-level estimates aggregated across observers. This residual variability is theoretically informative, because it highlights processes that shape memory success on individual occasions.

For the N400, such modulation may arise from trial-level variability in factors such as attention, momentary engagement, or individual differences in prior knowledge or familiarity with the content of the images—factors not captured by stimulus-level memorability, but that nonetheless shape semantic activation at encoding and influence later memory success. The LPC has more often been associated with processes that are accessible to awareness, including encoding fluency, recollective processing, and recognition confidence. Although we found that item-level memorability also accounts for a substantial portion of amplitude variance on the LPC, the remaining variance associated with subsequent memory may reflect encoding-related processes that contribute both to later recognition success and to participants’ subjective assessments of that success. Prior work has shown that people can make relatively accurate (although often biased) judgments about their own future memory performance (Dunlosky & Nelson, 1992; Schwartz, 1994), while having substantial difficulty judging the memorability of images themselves (Isola et al., 2014; Revsine & Bainbridge, 2025). Together, these findings suggest that memorability captures dominant, stimulus-driven contributions to encoding-related activity, while residual, trial-specific variance may support both successful memory formation and, perhaps particularly for the LPC, metacognitive evaluation.

## Conclusions

Our study replicates and extends prior evidence that highly memorable images evoke facilitated semantic activation and perceptual processing. At encoding, semantic activation appears to play a particularly important role in supporting memory strength, because it is sensitive to both memorability and subsequent memory performance, whereas perceptual processing is primarily tied to memorability. Critically, N400 responses were best predicted by memorability (i.e., stimulus-intrinsic factors that predict likelihood of recognition) when both memorability and downstream memory success were analyzed together. Taken together, these findings indicate that encoding-related neural activity linked to later memory success may largely reflect item-level properties rather than processes linked to successful in-the-moment encoding and that these properties ultimately drive differences in memory performance.

## Data Availability

Data or materials for the experiments are available upon request.

## Footnotes

1 However, we note that when we modeled memorability with continuous memorability scores, there was not a reliable effect on the N300. This could suggest that N300 amplitudes are sensitive at the extremes but are not truly graded with memorability, but more work is clearly needed to delineate the shape of the relationship between N300 amplitudes and memorability scores.

## Code availability

Analysis code of this study is available upon request.

## Declarations

### Open practices statement

Data or materials for the experiments are available upon request. The experiment was not preregistered.
